# Analysis of mechanical lysis paradigms for *Caenorhabditis elegans* protein extraction

**DOI:** 10.17912/micropub.biology.002356

**Published:** 2026-09-29

**Authors:** Sam K. Connors, Peter A. Kropp

**Affiliations:** 1 Biology, Kenyon College, Gambier, OH, United States

## Abstract

Extraction of proteins, mRNA, lipids, or any other biological material from *Caenorhabditis elegans* requires lysis of the cuticle. While many techniques have been utilized to this end, there are limited comparisons of mechanical lysis techniques individually or in combination. Therefore, we tested common mechanical lysis techniques to extract proteins from *C. elegans* samples. Due to cuticle structural differences, we compared these techniques with samples from both the L4 larval stage and day 1 adults. We found that mechanical lysis improves protein yield relative to no mechanical lysis and that most mechanical lysis techniques are comparable.

**Figure 1. Protein yield and quality analysis following mechanical lysis paradigms:** A) Schematic representation of different mechanical lysis paradigms and work flows. Plus signs indicate the inclusion of a technique. The snowflake represents the 5x freeze-thaw cycles for all samples prior to any mechanical lysis. Figure created in BioRender. B) Protein yield from L4 and D1A samples. Protein yield (ng) was normalized to the mass of the wet pellet (mg of sample). Each point represents an individual sample. *: p≤0.05 by One-Way ANOVA with Dunnet correction for multiple comparisons. Mean and standard deviation shown. n=5 per paradigm. C) Representative western blot analysis of TBA-1 with Ponceau S staining as a loading control. kD: kiloDalton.

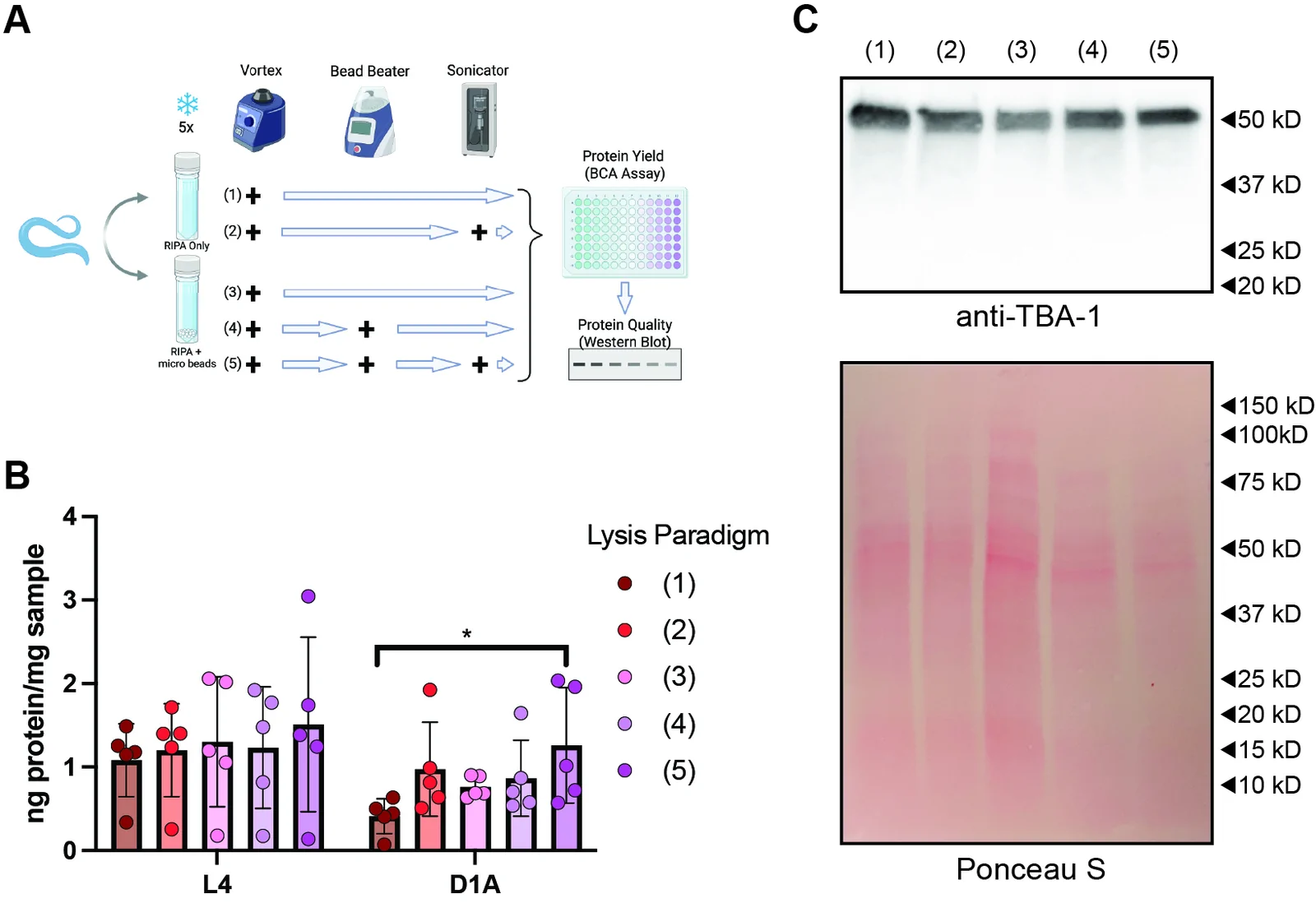



## Description

Analysis of biological material (e.g. proteins, mRNA, lipids) requires effective cell lysis, which is frequently predicated upon effective lysis of an intact tissue or organism. For intact organisms, lysis can be problematic if a cell wall or other structure poses as a barrier to lysing reagents. For example, lysis of *Saccharomyces cerevisiae* requires aggressive chemical or mechanical procedures due to the cell wall (Horvath & Riezman, 1994; Mukherjee et al., 2020) or lysis of dense and fibrotic tissue samples from mammals may require homogenization with a mechanical device (Ericsson & Nistér, 2010; Reis et al., 2025). *Caenorhabditis elegans* lysis poses a unique challenge due to the dense, fibrous cuticle that encapsulates the animal. This cuticle is largely impermeable making lysis with simple detergent-based lysis buffers ineffective. Many techniques have been utilized to disrupt the *C. elegans* cuticle including: freeze-thaw cycles in liquid nitrogen, mortar and pestle, bead beating, and sonication. Finding limited analyses of mechanical lysis techniques in the literature (Bhaskaran et al., 2011), we sought to directly test common lysis techniques, alone and in combination, that require varying degrees of material/instrument investment. We also sought to compare two ages of *C. elegans* , L4 and day 1 adult (D1A), since these are common ages for analysis and have appreciable differences in cuticle architecture and composition (Abete‐Luzi & Eisenmann, 2018; Liu et al., 1995; Sundaram & Pujol, 2024). We focused on protein extraction as our readout of lysis efficacy, but these results should be translatable to other materials including mRNA, metabolites, and lipids.

We age-synchronized wildtype *C. elegans* populations and collected them for protein analysis in RIPA ( r adio i mmuno p recipitation a ssay) buffer. All samples were flash frozen in liquid nitrogen and subjected to five cycles of freeze-thawing as a baseline. Following freeze-thawing, samples were grouped into one of five mechanical lysis paradigms ( Figure 1A ):

(1) Vortexing;

(2) Vortexing and sonication with a tip sonicator;

(3) Addition of 1.5 mm microbeads and vortexing;

(4) Addition of 1.5 mm microbeads, vortexing, and bead beating;

(5) Addition of 1.5 mm microbeads, vortexing, bead beating, and sonication with a tip sonicator.

We then pelleted the cellular debris, kept the soluble protein lysate, and analyzed it for both protein yield and protein quality (Figures 1B and C, respectively).

At the L4 larval stage, there were no significant differences in protein yield with any of the mechanical lysis paradigms: all samples yielded ~1 ng protein/mg wet pellet ( Figure 1B ). It should be noted, however, that yield was variable in all groups. At D1A, there were noticeable and significant differences between mechanical lysis paradigms ( Figure 1B ). Simply vortexing the sample in RIPA buffer, paradigm (1), resulted in the lowest protein yield (0.4120 ± 0.210 ng protein/mg pellet), and the most aggressive mechanical lysis, paradigm (5), yielded significantly more protein than group (1) but also had the highest variability of any group (1.261 ± 0.619 ng protein/mg pellet). The intermediate mechanical lysis techniques, groups (2), (3), and (4), all had comparable protein yields: 0.975 ± 0.562, 0.760 ± 0.127, and 0.866 ± 0.454 ng protein/mg pellet, respectively. Notably, tip sonicator use appears to result in the highest variation in protein yield (paradigms (2) and (5)).

Given that only D1A samples had appreciable differences in protein yield between mechanical lysis paradigms, we next tested if these lysis paradigms impacted protein quality. We speculated that more aggressive techniques (bead beating and sonication) could result in protein shearing or degradation which we analyzed with western blot analysis of the D1A samples, probing for TBA-1 , the *C. elegans* alpha-tubulin protein and a common gene used as a loading control. We did not observe any evidence of protein shearing or degradation as clean bands were observed in all groups ( Figure 1C ). Therefore, we conclude that the mechanical lysis paradigms tested here can improve protein yield with no evidence of decreased protein quality. It should be noted, however, that TBA-1 is a compact protein and larger, less structured proteins may be more impacted.

Our findings demonstrate that *C. elegans* age and mechanical lysis paradigm can both impact protein yield when making protein extracts. We were surprised that all lysis paradigms were equivalent at the L4 stage, but this finding could be due to the cuticle being thinner and less complex compared to D1A, rendering it easier to lyse (Cox et al., 1981). This explanation also supports the finding that protein yield was lower at D1A compared to L4 when using the same mechanical lysis paradigm, especially for paradigm (1) (L4: 1.082 μg/mg; D1A: 0.4120 μg/mg). Although we did not test dauer larvae, this stage has a uniquely impermeable cuticle (Androwski et al., 2017; Cassada & Russell, 1975; Cox et al., 1981), which may be harder to lyse than the ages analyzed here.

Given the similar efficacy of mechanical lysis paradigms at D1A, each investigator should feel confident choosing the method that best suits their lab and available instrumentation. However, we do note that variability in protein yield was smallest for samples that were only vortexed, with or without microbeads. Investigators should be mindful that the more aggressive techniques also generate more bubbly/agitated samples which appear to impact sample recovery and increased protein yield variability. This tradeoff must be considered when deciding the best technique to use. We also note the limitation of this comparative study in that we only assessed protein yield. We predict that the trends observed in our results will hold for extraction of other biological materials, but quantitative comparisons should be considered by the investigator.

## Methods

*C. elegans* maintenance

*C. elegans* were maintained on *Escherichia coli* strain OP50 at 20°C on MYOB plates following standard practices (Brenner, 1974).

Sample preparation

Synchronous populations of *C. elegans* were generated by sodium hypochlorite synchronization. Harvested embryos were hatched overnight in S-Basal and plated as L1s. All samples utilized 500 animals per plate/sample. L4 samples were harvested 44-48 hours after plating and D1A samples were harvested 72 hours after plating.

Animals were washed from plates with M9 and collected in 1.5 mL tubes. Samples were washed 3 times with M9 and a final wash with Milli-Q water. In the final wash, tubes were placed on ice for 5 seconds to allow pellet consolidation and removal of additional supernatant. Wet pellets were weighed to determine the sample mass. 150 μL RIPA (ThermoFisher Scientific #J63306-AK) with 1X HALT Protease and Phosphatase Inhibitor Cocktail (ThermoFisher Scientific #78440) was added and samples were immediately frozen in liquid nitrogen. Samples were subjected to 5 freeze-thaw cycles of thawing on ice and refreezing in liquid nitrogen.

Mechanical lysis techniques

Vortex: Samples were vortexed continuously for 1 minute.

Sonication: Samples were sonicated for 5 x 1 second pulses at 50% amplitude with a tip sonicator (QSonica Q125 Ultrasonic Processor), rested on ice for 5 minutes, and then the sonication was repeated.

Microbeads: Samples were processed in 2.0 mL screwcap tubes loaded with 1.5 mm zirconium beads (Benchmark Scientific #D1032-15). 1.5 mm microbeads were chosen so that they were too large to fit in the opening of a pipet tip, but still offer a large surface area for sample contact.

Bead beating: Samples with microbeads (above) were shaken 2 x 1 minute at 4,000 rpm with a Beadbug Microtube Homogenizer (Benchmark Scientific #D1030) at 4°C. Samples were rested on ice for 5 minutes between rounds of beating.

Protein extraction and quantification

Following mechanical lysis, samples were transferred to a 1.5 mL tube (if bead beating had occurred) and cellular debris was pelleted by centrifugation for 15 minutes at 14,000 rpm at 4°C. The resulting supernatants were transferred to fresh tubes and protein concentration was determined using a Pierce BCA Protein Assay (ThermoFisher Scientific #PI23227). If not used immediately, samples were stored at -80°C. Samples were limited to one freeze-thaw cycle after this stage.

Protein yield was normalized to the mass of the wet pellet.

Western blotting

Western blot analysis was performed in triplicate. For each sample, 10 μg protein was prepared in 30 μL with 5 mM DTT and 1X Laemmeli Sample Buffer (Bio-Rad #1610747). Samples were boiled for 10 minutes at 95°C, loaded into Any kD Mini-PROTEAN TGX Stain-Free Gels (Bio-Rad #4569033) with the Precision Plus Protein WesternC standard (Bio-Rad #1610399), and run with Tris-Glycine-SDS buffer (Bio-Rad 1610732) at 200 V. Proteins were transferred to 0.2 μm nitrocellulose membranes (Bio-Rad 1704158) with a Trans-Blot Turbo Transfer System (Bio-Rad #1704150). Membranes were stained with 0.5% Ponceau S (w:v) in 5% glacial acetic acid for 5 minutes at room temperature, destained with 0.1% (w:v) NaOH, and rinsed with distilled water. Membranes were blocked with Everyblot Blocking Buffer (Bio-Rad #12010020) and incubated overnight at 4°C with the Mouse anti-alpha tubulin primary antibody (DSHB #12G10; 1:2,500) in 3% (w:v) BSA in TBST. Membranes were washed 5 x 5 minutes with TBST and incubated for 2 hours at room temperature with the HRP Goat anti-Mouse secondary antibody (Proteintech #SA00001-2; 1:10,000) and Streptactin (Bio-Rad #1610381; 1:50,000) in 3% BSA. Membranes were washed 5 x 5 minutes with TBST and developed with Clarity Western ECL substrate (Bio-Rad #1705060) and imaged with a ChemiDoc MP imager (Bio-Rad).

Statistical analysis

Sample sizes and statistical tests are reported in the figure legend. Statistical analysis was performed with GraphPad Prism version 11.0.0.

## Reagents

**Table:** 

**Strain**	**Genotype**	**Source**
N2	WT *C. elegans*	*Caenorhabditis* Genetics Center
**Antibody**	**Species and reactivity**	**Source**
Anti-Alpha Tubulin	Mouse, monoclonal	Developmental Studies Hybridoma Bank
